# Supplementary material for: Cell-mechanical parameter estimation from 1D cell trajectories using simulation-based inference
Source: PLoS One. 2025 Jun 18;20(6):e0310669. doi: 10.1371/journal.pone.0310669 (PMC12176162; doi:10.1371/journal.pone.0310669)
Supplement: S1 File — The SI contains information about the inference of 10 free parameters, a simplification of the original biophysical model, the quality of the neural density estimator (NDE) and the effect of additional information on the performance of SBI. (PDF) [file pone.0310669.s001.pdf]

Supporting information

Cell-mechanical parameter estimation from 1D cell trajectories using simulation-based inference

Johannes C. J. Heyn[1,¶] , Miguel Atienza Juanatey[1,¶] , Martin Falcke[2], Joachim O. Rädler[1,*]

[1] Fakultät für Physik, Ludwig-Maximilians-Universität München (LMU), Munich, Germany

[2] Max Delbrück Center for Molecular Medicine in the Helmholtz Association, Berlin, Germany

[*]Correspondence: raedler@lmu.de

[¶]These authors contributed equally to this work.

Inference of 10 free parameters

Initially, we tried to infer 10 free parameters of the biophysical model, namely {E, L_0, V_e^0, k_{on}, K_{off}, v_{rc}, k_{max}, ζ_{max}, b, B} (for a description of the parameters, see Tab. 1). To this end we trained an NDE as described above but with the goal of calculating 10 posterior distributions instead of 5, as in the later version of our NDE. The result of the inference is shown in Fig S1. The plots on the diagonal show the posterior distribution for each individual parameter $p(\theta_i|x)$, while the plots in the right-hand corner show the distribution for each pair of parameters $p(\theta_{ij}|x)$. Vertical gray lines in the plots on the diagonal and white crosses in the plots on the off-diagonal represent the values that were used for the simulated trajectory. While the peak of the posterior distributions is close to the true value for most parameters, the distributions are smeared out across the range of the prior distribution which means that the NDE can't infer the true parameter set precisely. Additionally, the shape of the 2D posterior distributions in the off-diagonal plots exhibit small intrinsic correlations between different parameters. Diagonal shapes in the plots of paired distributions, such as for the actin retrograde flow velocity V_e^0 vs the critical actin retrograde flow velocity v_{rc} and the ligand density B vs the effective E-modulus E, indicate correlations of parameters of the biophysical model. A rising shape of the paired distribution is a sign of a positive correlation, such as for V_e^0 vs v_{rc} and B vs E, while a falling shape such as for k_{max} vs V_e, is a hallmark of a negative correlation. The correlations between parameters imply that we cannot accurately determine the values of individual parameters independently. We see this for example in the shape of $p(k_{max}, V_e|\mathbf{x})$: The negative correlation between k_{max} and V_e tells us that an increase in one is equivalent to a decrease in the other, so that we cannot determine k_{max} and V_e simultaneously.

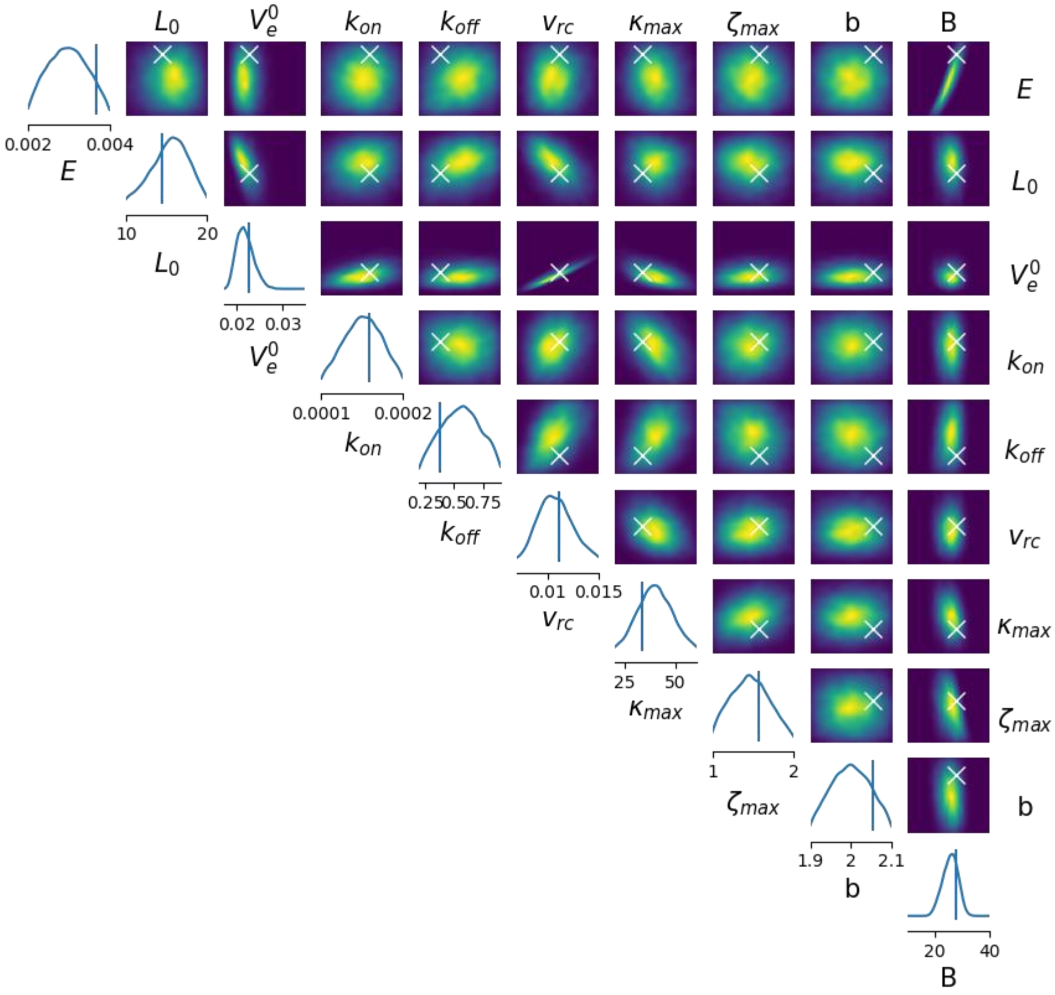

Fig S1 Inference of 10 free parameters leads to loss of identifiability. The posterior probability p(**θ**|**x**) is inferred by a neural density estimator that was learnt to infer 10 free parameters. The plots on the diagonal show the posterior distribution for each individual parameter $p(\theta_i|x)$, while the plots in the right-hand corner show the distribution for each pair of parameters $p(\theta_{ij}|x)$. Vertical gray lines in the plots on the diagonal and white crosses in the plots on the off-diagonal represent the values that were used for the simulated trajectory. The posterior distributions are smeared out across the range of the prior distribution which means that the NDE can’t infer the true parameter set precisely.

Simplification of the biophysical model

The size of the latent space required a reduction of our original model for a sensible parameter inference. We reduced the number of parameters by rescaling and simplifying the model. Fibronectin density B as external parameter is assumed to be constant because the FN density on the 1D lanes was kept constant. The vectorial sum of the actin retrograde flow v_r and the cell's velocity v are equal to the net networking extension rate V_e.

$$v_{r_f} + v_f = V_e^0 \exp\left(\frac{-aF_f}{N}\right) - k^- \quad (A1)$$

$$v_{r_b} - v_b = V_e^0 \exp\left(\frac{-aF_b}{N}\right) - k^- \quad (A2)$$

The coefficient a

$$a = gd/k_b T \quad (A3)$$

incorporates a geometrical component g that results from averaging the orientation of the filaments within the network, the increase in length d=2.7 nm contributed by each actin monomer to the filament, and the thermal energy $k_b T$. We assume that the force dependence of the actin polymerization is negligible, so that the actin network extends at the constant rate of Ve0. We also eliminate the depolymerization rate k- which additionally reduces the computational cost of simulations.

$$v_{r_f} + v_f = V_e^0 \quad (A4)$$

$$v_{r_b} - v_b = V_e^0 \quad (A5)$$

Changes in the protrusion length L arise from the difference of the velocities of cell edge and nucleus.

$$\frac{dL_f}{dt} = v_f - v_c \quad (A6)$$

$$\frac{dL_b}{dt} = -v_b + v_c \quad (A7)$$

Integrin signaling is non-linear. It's governed by the following equations:

$$\frac{d\kappa_f}{dt} = k_{on}(\kappa_{max} - \kappa_f) - k_{off} e^{-\frac{v_{r_f}}{v_{slip}}} \kappa_f \quad (A8)$$

$$\frac{d\kappa_b}{dt} = k_{on}(\kappa_{max} - \kappa_b) - k_{off} e^{-\frac{v_{r_b}}{v_{slip}}} \kappa_b \quad (A9)$$

Note that we renamed c_1 as k_{on}, c_2 as k_{off} and c3 to v_{slip} for clarity compared to Amiri et al.

Integrin signaling is affected by the fibronectin density of the substrate. The upper value of κ and ζ is determined by the Hill functions in equations S17 and S18 of Amiri et al. This relation is reflected by the adhesion velocity relation. We therefore use the adhesion velocity relation to fix the four parameters entering the Hill functions: n_κ, n_ζ, K_κ, K_ζ. We furthermore assume $\zeta_0 = \kappa_0 = 0$ as their values used in Amiri et al. are negligibly small and therefore have a negligible effect on simulated trajectories.

We rescale the force balance (eqs. S2 and S4, Amiri et al.) by V_e^0 and L and arrive at the following equation for the cell's edges:

$$\kappa V_{\mathrm{e}}^0 \frac{v_{\mathrm{r}}}{V_{\mathrm{e}}^0} - L_0 E(\frac{L_{\mathrm{f}}}{L_0} - 1) - \zeta_{\mathrm{f}} V_{\mathrm{e}}^0 \frac{v}{V_{\mathrm{e}}^0} = 0 \tag{A10}$$

We define

$$\tilde{v}_{\mathrm{r}} \equiv \frac{v_{\mathrm{r}}}{V_{\mathrm{e}}^0} \tag{A11}$$

$$\tilde{L}_{\mathrm{f}} \equiv \frac{L_{\mathrm{f}}}{L_0} \tag{A12}$$

$$\tilde{E} \equiv E L_0 \tag{A13}$$

and rescale κ, ζ in eq. A10 by the factor $E\frac{L_0}{v_{\mathrm{e}}^0}$

$$\frac{EL_0}{V_{\mathrm{e}}^0} \tilde{\kappa} V_{\mathrm{e}}^0 \tilde{v}_{\mathrm{r}} - \tilde{E}(\tilde{L} - 1) - \frac{EL_0}{V_{\mathrm{e}}^0} \tilde{\zeta} V_{\mathrm{e}}^0 \tilde{v} = 0 \tag{A14}$$

$$\tilde{E}\tilde{\kappa}\tilde{v}_{\mathrm{r}} - \tilde{E}(\tilde{L} - 1) - \tilde{E}\tilde{\zeta}\tilde{v} = 0 \tag{A15}$$

which yields the following formulation of the force balance

$$\tilde{\kappa}_{\mathrm{f}} \tilde{v}_{\mathrm{r_f}} - \tilde{L}_{\mathrm{f}} + 1 - \tilde{\zeta}_{\mathrm{f}} \tilde{v}_{\mathrm{f}} = 0 \tag{A16}$$

$$-\tilde{\kappa}_{\mathrm{b}} \tilde{v}_{\mathrm{r_b}} + \tilde{L}_{\mathrm{b}} - 1 + \tilde{\zeta}_{\mathrm{b}} \tilde{v}_{\mathrm{b}} = 0 \tag{A17}$$

$$\tilde{L}_{\mathrm{f}} - \tilde{L}_{\mathrm{b}} - \tilde{\zeta}_{\mathrm{c}} \tilde{v}_{\mathrm{c}} = 0 \tag{A18}$$

with

$$\tilde{v}_{\mathrm{r_f}} + \tilde{v}_{\mathrm{f}} = 1 \tag{A19}$$

$$\tilde{v}_{\mathrm{r_b}} + \tilde{v}_{\mathrm{b}} = 1 \tag{A20}$$

We now rescale eqs. A6 and A7 by introducing

$$\tau \equiv t\frac{V_{\mathrm{e}}^0}{L_0} \tag{A21}$$

$$L_0 \frac{\mathrm{d}\tilde{L}}{\mathrm{d}t} = v_{\mathrm{f}} - v_{\mathrm{c}} \tag{A22}$$

$$\rightarrow \frac{L_0}{V_{\mathrm{e}}^0} \frac{\mathrm{d}\tilde{L}}{\mathrm{d}(\tau \frac{L_0}{V_{\mathrm{e}}^0})} = \tilde{v}_{\mathrm{f}} - \tilde{v}_{\mathrm{c}} \tag{A23}$$

which yields

$$\frac{\mathrm{d}\tilde{L}_{\mathrm{f}}}{\mathrm{d}\tau} = \tilde{v}_{\mathrm{f}} - \tilde{v}_{\mathrm{c}} \tag{A24}$$

$$\frac{\mathrm{d}\tilde{L}_{\mathrm{b}}}{\mathrm{d}\tau} = -\tilde{v}_{\mathrm{b}} + \tilde{v}_{\mathrm{c}} \tag{A25}$$

and for the κ-dynamics

$$\frac{V_{\mathrm{e}}^{0}}{L_{0}}\frac{\mathrm{d}\kappa}{\mathrm{d}\tau}=\mathrm{k}_{\mathrm{on}}\kappa_{\mathrm{max}}-\kappa(\mathrm{k}_{\mathrm{on}}+\mathrm{k}_{\mathrm{off}}e^{-\frac{v_{\mathrm{r}}}{v_{\mathrm{slip}}}}) \tag{A26}$$

we rescale κ and κ_{max} again with τ

$$\frac{v_{\mathrm{e}}^{0}}{L_{0}}\frac{\mathrm{d}\tilde{\kappa}}{\mathrm{d}\tau}=\mathrm{k}_{\mathrm{on}}\tilde{\kappa}_{\mathrm{max}}-\tilde{\kappa}(\mathrm{k}_{\mathrm{on}}+\mathrm{k}_{\mathrm{off}}e^{-\frac{v_{\mathrm{r}}}{v_{\mathrm{slip}}}}) \tag{A27}$$

Finally, we rescale $\tilde{\mathrm{k}}_{\mathrm{on}}$ and $\tilde{\mathrm{k}}_{\mathrm{off}}$

$$\tilde{\mathrm{k}}_{\mathrm{on}}=\mathrm{k}_{\mathrm{on}}\frac{L_{0}}{v_{\mathrm{e}}^{0}} \tag{A28}$$

$$\tilde{\mathrm{k}}_{\mathrm{off}}=\mathrm{k}_{\mathrm{off}}\frac{L_{0}}{v_{\mathrm{e}}^{0}} \tag{A29}$$

to

$$\frac{\mathrm{d}\tilde{\kappa}}{\mathrm{d}\tau}=\tilde{\mathrm{k}}_{\mathrm{on}}\tilde{\kappa}_{\mathrm{max}}-\tilde{\kappa}(\tilde{\mathrm{k}}_{\mathrm{on}}+\tilde{\mathrm{k}}_{\mathrm{off}}e^{-\frac{\tilde{v}_{\mathrm{r}}}{\tilde{v}_{\mathrm{slip}}}}) \tag{A30}$$

which yields

$$\frac{\mathrm{d}\tilde{\kappa}_{\mathrm{f}}}{\mathrm{d}\tau}=\tilde{\mathrm{k}}_{\mathrm{on}}\tilde{\kappa}_{\mathrm{max}}-\tilde{\kappa}_{\mathrm{f}}(\tilde{\mathrm{k}}_{\mathrm{on}}+\tilde{\mathrm{k}}_{\mathrm{off}}e^{-\frac{\tilde{v}_{\mathrm{r_f}}}{\tilde{v}_{\mathrm{slip}}}}) \tag{A31}$$

$$\frac{\mathrm{d}\tilde{\kappa}_{\mathrm{b}}}{\mathrm{d}\tau}=\tilde{\mathrm{k}}_{\mathrm{on}}\tilde{\kappa}_{\mathrm{max}}-\tilde{\kappa}_{\mathrm{b}}(\tilde{\mathrm{k}}_{\mathrm{on}}+\tilde{\mathrm{k}}_{\mathrm{off}}e^{-\frac{\tilde{v}_{\mathrm{r_b}}}{\tilde{v}_{\mathrm{slip}}}}) \tag{A32}$$

Altogether the rescaled system is given by

$$\tilde{\kappa}_{\mathrm{f}}\tilde{v}_{\mathrm{r_f}}-\tilde{L}_{\mathrm{f}}+1-\tilde{\zeta}_{\mathrm{f}}\tilde{v}_{\mathrm{f}}=0 \tag{A33}$$

$$-\tilde{\kappa}_{\mathrm{b}}\tilde{v}_{\mathrm{r_b}}+\tilde{L}_{\mathrm{b}}-1+\tilde{\zeta}_{\mathrm{b}}\tilde{v}_{\mathrm{b}}=0 \tag{A34}$$

$$\tilde{L}_{\mathrm{f}}-\tilde{L}_{\mathrm{b}}-\tilde{\zeta}_{\mathrm{c}}\tilde{v}_{\mathrm{c}}=0 \tag{A35}$$

$$\tilde{v}_{\mathrm{r_f}}+\tilde{v}_{\mathrm{f}}=1 \tag{A36}$$

$$\tilde{v}_{\mathrm{r_b}}-\tilde{v}_{\mathrm{b}}=1 \tag{A37}$$

$$\frac{\mathrm{d}\tilde{L}_{\mathrm{f}}}{\mathrm{d}\tau}=\tilde{v}_{\mathrm{f}}-\tilde{v}_{\mathrm{c}} \tag{A38}$$

$$\frac{\mathrm{d}\tilde{L}_{\mathrm{b}}}{\mathrm{d}\tau}=-\tilde{v}_{\mathrm{b}}+\tilde{v}_{\mathrm{c}} \tag{A39}$$

$$\frac{\mathrm{d}\tilde{\kappa}_{\mathrm{f}}}{\mathrm{d}\tau}=\tilde{\mathrm{k}}_{\mathrm{on}}\tilde{\kappa}_{\mathrm{max}}-\tilde{\kappa}_{\mathrm{f}}(\tilde{\mathrm{k}}_{\mathrm{on}}+\tilde{\mathrm{k}}_{\mathrm{off}}e^{-\frac{\tilde{v}_{\mathrm{r_f}}}{\tilde{v}_{\mathrm{slip}}}}) \tag{A40}$$

$$\frac{\mathrm{d}\tilde{\kappa}_{\mathrm{b}}}{\mathrm{d}\tau}=\tilde{\mathrm{k}}_{\mathrm{on}}\tilde{\kappa}_{\mathrm{max}}-\tilde{\kappa}_{\mathrm{b}}(\tilde{\mathrm{k}}_{\mathrm{on}}+\tilde{\mathrm{k}}_{\mathrm{off}}e^{-\frac{\tilde{v}_{\mathrm{r_b}}}{\tilde{v}_{\mathrm{slip}}}}) \tag{A41}$$

The original system had 9 internal parameters:

$$\{E, L_0, v_e^0, \zeta, b, \mathrm{k_{on}}, \mathrm{k_{off}}, \kappa_{\mathrm{max}}, v_{\mathrm{slip}}\}. \tag{A42}$$

The parameters in the rescaled model are

$$\{\tilde{\zeta}_f, \tilde{\zeta}_b, \tilde{\zeta}_c, \tilde{\mathrm{k}}_{\mathrm{on}}, \tilde{\mathrm{k}}_{\mathrm{off}}, \tilde{\kappa}_{\mathrm{max}}, \tilde{v}_{\mathrm{slip}}\} \tag{A43}$$

with $\zeta_f = \zeta_b$, $\zeta_c = b\zeta_f$ reducing the set to 6 parameters,

$$\{\tilde{\zeta}, b, \tilde{\mathrm{k}}_{\mathrm{on}}, \tilde{\mathrm{k}}_{\mathrm{off}}, \tilde{\kappa}_{\mathrm{max}}, \tilde{v}_{\mathrm{slip}}\} \tag{A44}$$

which are given by

$$\{\zeta_f \frac{V_e^0}{EL_0}, b, \mathrm{k_{on}}\frac{L_0}{V_e^0}, \mathrm{k_{off}}\frac{L_0}{v_e^0}, \kappa_{\mathrm{max}}\frac{V_e^0}{EL_0}, \frac{v_{\mathrm{slip}}}{V_e^0}\}. \tag{A45}$$

Since we do not assume the dimensionless parameter $b = \zeta_c/\zeta_f$, which is the contribution of the cell's body to the drag compared to the contribution of the protrusions, to be cell type or cell state specific, we keep it fixed at b=3. Hence, the simplification of the model results in 5 variable parameters that sufficiently describe the system's dynamics. As the 5 targets of the inference procedure, we choose parameters that provide the best biological insight, either by being easily interpretable or experimentally accessible: $\{L_0, V_e^0, k_{on}, k_{max}, v_{slip}\}$.

Quality of the NDE

A good posterior estimator $p(\theta|x)$ should satisfy two conditions. First it should have a high predictive power: it should assign much higher probability to the true parameter value than the prior does

$p(\theta_{true}|x) >> p(\theta)$.

Second, a good posterior should be well calibrated: it should be unbiased, and it should be neither overconfident nor underconfident.

We introduce the metric $L_{i,j} = \sum_{n=1}^{N} p(\theta^i_n, \theta^j_n|x) / p(\theta^i_n, \theta^j_n)$ as a measure of the accuracy of the inference procedure for each pair of parameters, where θ^i_n is the i-th component of the nth true parameter. The resulting L_{ij} is shown in Fig 4 and shows which parameters are best inferred by the trained NDE. In particular, we can also directly see how correlations between different parameters lead to a better inference of joint probability distributions than for individual parameters: $L_{ij} > L_{ii}$ and $L_{ij} > L_{jj}$.

We then check whether our posterior is well calibrated using simulation-based calibration (SBC) (65). SBC is capable of revealing two main problems in a neural posterior density estimator (NDE):

1. Biases in the inferred posterior: SBC reveals systematic over or underestimation of any specific parameter.
2. Posterior uncertainty. SBC also allows checking for an overconfident or underconfident posterior. A good brief explanation is provided in https://www.mackelab.org/sbi/tutorial/13_diagnostics_simulation_based_calibration/

The main idea behind SBC goes as follows:

1. Run N simulations
2. For each simulation, sample L samples from the posterior $\{\theta_1, \ldots, \theta_L\} \sim p(\theta|x)$
3. Compute the rank statistic of $\{\theta_1, \ldots, \theta_L\}$ (see Talts et al. [65] for details) for each simulation

4. Increment the histogram for all N simulations

The rank statistics for the N_{sim} simulations should be uniformly distributed. Any deviations from uniformity show an uncalibrated posterior. An asymmetric distribution of the rank statistics shows a bias in the given parameter. A U-shape shows an overconfident posterior. An ∩-shaped distribution shows an under confident posterior. Examples of the rank statistics for 1023 simulations (N=23) are displayed in S2 Fig.

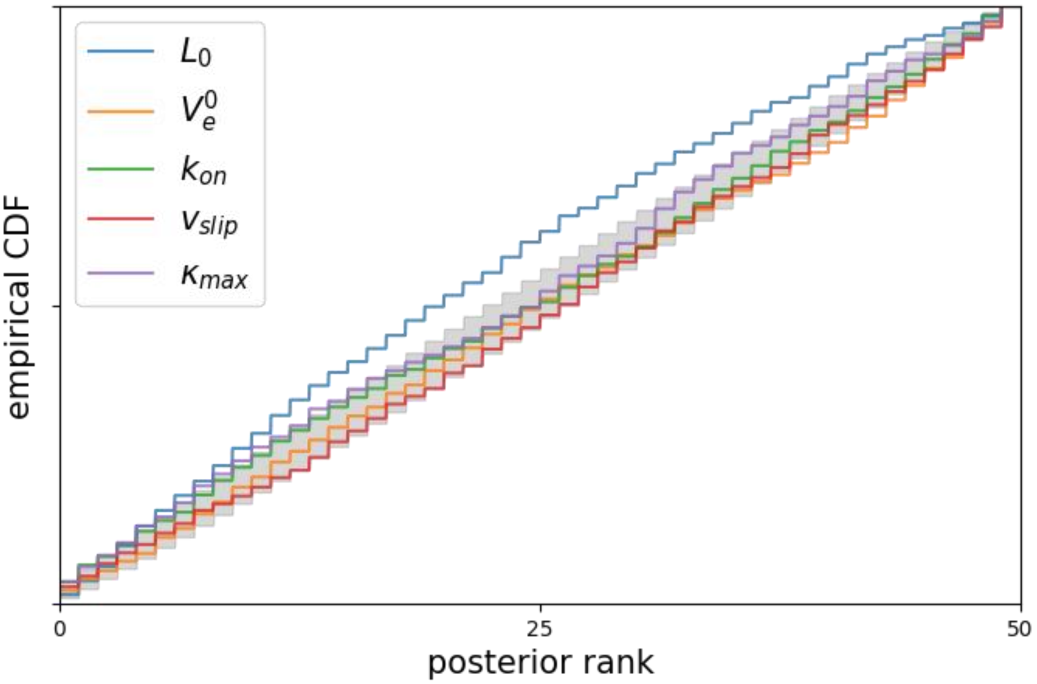

Fig S2 Quality of the NDE Examples of the rank statistics for 1023 simulations (N=23). The rank statistics for the N_{sim} simulations should be uniformly distributed and fall within the gray area. The parameters V_e^0, k_{on}, v_{slip} and κ_{max} can be considered as being well calibrated while the posterior estimation for L_0 is somewhat under-confident.

Additional information improves the performance of SBI.

The experiments performed in this work track three variables over time: front, back, and nuclear position. In this section we investigate how additional measurements would improve the precision of the model-based characterization of single cells. Training the NDE in Fig 4 required a parameter set θ and a corresponding simulation x = (front, back, nucleus). In the following we expanded x with other measurable variables and investigated the effect these additional variables had on the NDE's performance. To this end we include values used for simulations of those variables that could potentially be measured into the SBI input. The results are shown in S3 Fig. We start by adding the actin retrograde flow at the front and back of the cell, so that x = (front, back, nucleus, $v_{r,f}$, $v_{r,b}$), see S3(b) Fig. This leads to the posterior distribution for v_{slip} being more peaked. If instead κ_f and κ_b the adhesion dynamics are directly accessible to the NDE, not only v_{slip} but also the actin network extension rate V_e^0 can be estimated with more confidence, see S3(c) Fig. If information characterizing both the retrograde flow

and the adhesion dynamics are available to the NDE, all remaining parameters can be inferred with great confidence, see S3(d) Fig.

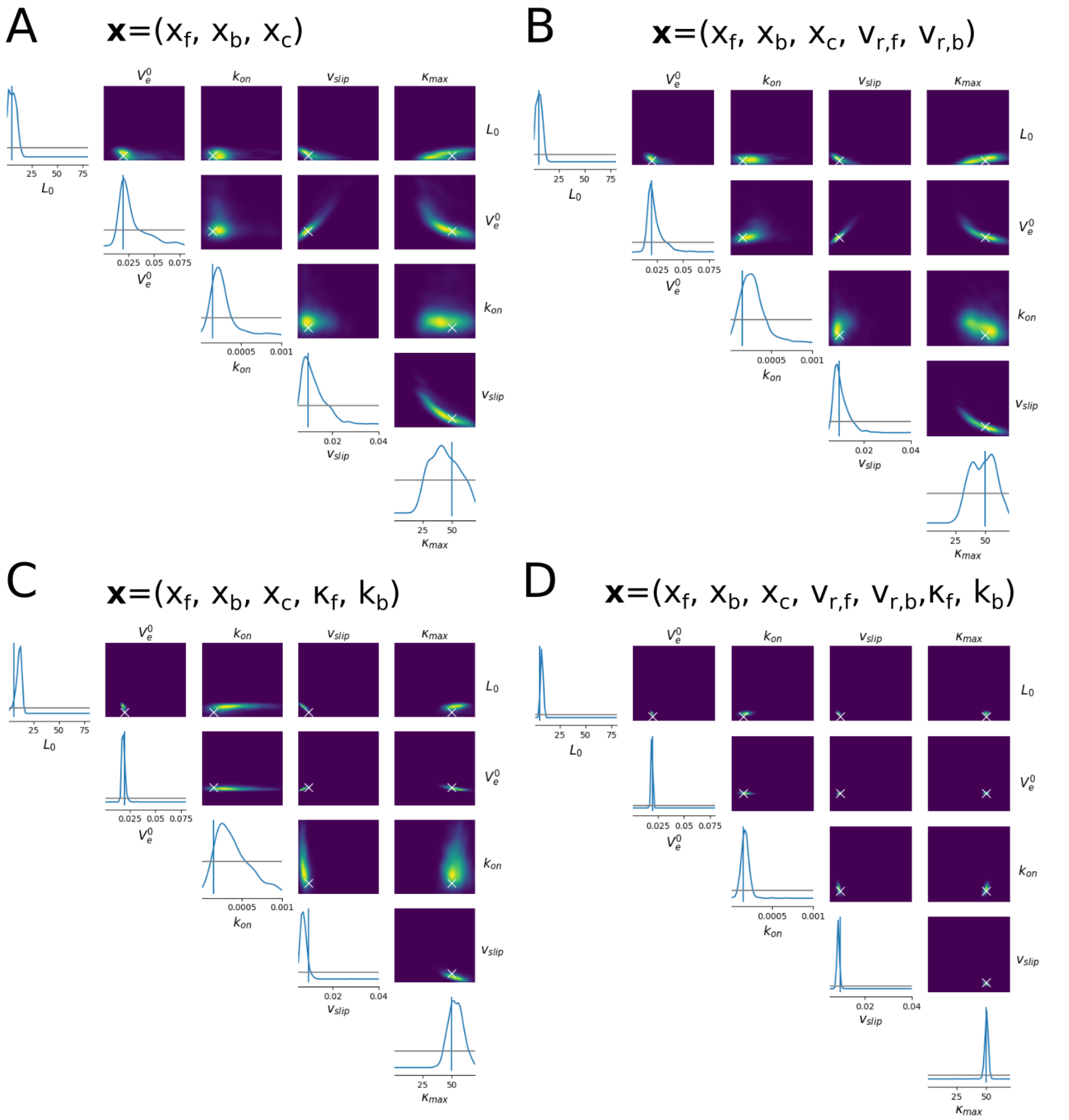

Fig S3 New variables improve SBI performance. The posterior p(θ|x) was inferred by our trained NDPE. (a) We compare a posterior trained with the three cellular positions as input. (b) Here, the input variables contain not only the cellular positions but also the actin retrograde flows $v_{r,f}$, $v_{r,b}$. (c) This plot depicts a posterior trained on the cellular positions plus the adhesion dynamics κ_f and κ_b. (d) Finally, the posterior if the input contains the cellular positions, the actin retrograde flows and the adhesion dynamics. The sharpening of the posterior estimator with the addition of observed variables suggests

that one could characterize migrating cells much more precisely by adding further readouts to the experimental tracking.
